# Broad-spectrum antimicrobial activity of a citric acid–phenolic formulation in animal feed matrices

**DOI:** 10.3389/fmicb.2026.1822735

**Published:** 2026-05-04

**Authors:** David Díez Arias, Anna Tesouro Rodriguez, Dongming Zhang, Amin Yousefvand

**Affiliations:** 1 Technical Department, Biovet, S.A., Constantí, Spain; 2 Department of Microbiology, Faculty of Agriculture and Forestry, University of Helsinki, Helsinki, Finland; 3 Helsinki Institute of Sustainability Science (HELSUS), University of Helsinki, Helsinki, Finland

**Keywords:** animal feed, antimicrobial, board spectrum, natural preservatives, one health

## Abstract

Microbial contamination in animal feed undermines livestock safety and storage stability, driving a critical demand for potent natural preservatives. This study investigated the antimicrobial efficacy of Alquermold Natural Plus L (AMNL), a formulation combining citric acid and bioactive monoterpenes, within compound feed and ground corn matrices. AMNL, applied at a dosage of 0.5 L/ton, was evaluated against a commercial benchmark at 2 L/ton. Both treatments were tested against key bacterial pathogens (*Escherichia coli*, *Salmonella Typhimurium*, and *Pseudomonas aeruginosa*) and fungal contaminants (*Aspergillus niger*, *Fusarium oxysporum*, and *Rhizopus nigricans*). Substrates were standardized to a contamination level of 10^6^ CFU/g, with microbial reduction measured at 24 h and 7 days. The results revealed that AMNL consistently achieved greater reductions in bacterial and fungal populations than the competitor (a commercial feed preservative based primarily on organic acids), despite being applied at one-quarter of the inclusion rate. Antimicrobial performance improved over storage duration, particularly against bacterial taxa, while maintaining consistent efficacy across diverse microbial species and feed matrices. Linear mixed-effects modeling confirmed significant main effects for both treatment and time. These findings demonstrate that the formulation combining citric acid and monoterpenes provides effective microbial control, supporting its application as a high-performance natural alternative to conventional synthetic feed preservatives.

## 1 Introduction

The quality of animal feed is significantly compromised by microbiological contamination, which leads to both safety risks for livestock and substantial economic losses (Tiwari et al., 2006; Davidson et al., 2012). Finished products, such as ground corn and compound feed, are susceptible to a wide range of bacterial pathogens, including *Salmonella Typhimurium*, *Escherichia coli*, and *Staphylococcus aureus*, as well as fungal spoilage driven by species such as *Aspergillus niger* and *Fusarium* (ICMSF, 1996; Tsai et al., 2000; Singh et al., 2002; Stopforth et al., 2005; Efenberger-Szmechtyk et al., 2021). Ensuring the stability of these ingredients is challenging, as their varying nutritional compositions and moisture levels provide fertile ground for microbial proliferation and the formation of resistant biofilms (Tiwari et al., 2006; Kifer et al., 2016; Bouarab-Chibane et al., 2018).

Traditionally, the industry has relied on chemical preservatives, specifically organic acids such as acetic, citric, lactic, formic, and propionic acids (Silva and Lidon, 2016). The effectiveness of these weak acids is highly pH-dependent, as they are most potent in their undissociated form (Doores, 2005). In this state, they can penetrate the microbial cytoplasmic membrane, subsequently dissociating within the cell to acidify the cytoplasm and disrupt essential metabolic processes (Doores, 2005). However, the use of synthetic additives is increasingly restricted due to regulatory changes and concerns regarding health and safety, including corrosive properties, predominantly bacteriostatic modes of action with limited efficacy, and potential side effects, such as the formation of carcinogenic nitrosamines and the contribution to antimicrobial resistance (Silva and Lidon, 2016; Inetianbor et al., 2015).

This shift has driven intensive research into natural plant-based antimicrobials, particularly monoterpenoid phenols such as thymol (Memar et al., 2017; FDA, 2016; European Commission, 1999). These phytochemicals, primarily found in the *Lamiaceae* family, exhibit a broad spectrum of activity against bacteria, yeasts, and fungi (Vila, 2002; Suntres et al., 2015). Their primary mechanism of action involves interacting with the lipid bilayer of the cytoplasmic membrane, increasing its fluidity and permeability, which leads to the leakage of vital intracellular materials, such as potassium ions, ATP, and nucleic acids (Ultee et al., 1999; Martínez-Romero et al., 2007).

Despite their potency, the application of individual monoterpenes is often limited by high volatility, low water solubility, and pungent flavor profiles (Wang and Wu, 2021). A promising solution is the application of antimicrobial synergy through “hurdle technology,” where combinations of different agents achieve higher efficacy at lower inclusion rates (Lidon and Silvestre, 2008). Research suggests that combining organic acids with phenolic compounds can potentiate activity; for instance, citric acid can facilitate the entry of phenols into the cell, allowing for more efficient disruption of the membrane and respiratory chain (Vasseur et al., 1999; Mączka et al., 2023).

While the properties of individual natural preservatives are well-documented, there is a lack of data regarding their long-term effectiveness in complex feed environments (Oulahal and Degraeve, 2022). Consequently, this study aims to evaluate the microbicidal efficacy of Alquermold Natural Plus L (AMNL), a synergistic blend of citric acid and phenolic monoterpenes, in reducing microbial loads in ground corn and compound feed. By comparing its performance with that of a commercial competitor (preservative based primarily on organic acids) over a 7-day period, this research seeks to establish a reliable, natural approach to feed storage and preservation.

## 2 Materials and methods

### 2.1 Microbes and substrates

The bacterial strains used in this study were *Clostridium perfringens* ATCC 3642, *Escherichia coli* Nissle 1917*, Salmonella enterica serovar Typhimurium* ATCC 23591, and *Pseudomonas aeruginosa* ATCC 10145. Fungal strains included *Aspergillus niger* 495*, Rhizopus nigricans* FBCC2513*, Fusarium oxysporum* f.sp. *cubense*. AMNL contained 3.5% isopropyl-methylphenol (thymol), 1% citric acid, and 0.4% cineole + cimenol ring (from *Eucalyptus globulus*). The competitor was composed of 40% ammonium dipropionate, 9% formic acid, and 33% formaldehyde. Sterilized compound feed and ground corn were used to test the antimicrobial capacity of the treatments. The composition of the compound feed (Zooplus, Finland) was: 14.3% protein, 3.7% fat, 2% fiber, 8.8% ash, and 0.16% phosphorus. Ground corn was purchased from the local market in Finland; its nutrient compositions were protein 8.8%, fat 1.1%, fiber 5%, and carbohydrates 73.8%.

Violet Red Bile Agar (VRBA) media was used for the enumeration of the *E. coli* strain. Xylose lysine deoxycholate (XLD) agar medium was applied for Salmonella enumeration. *Pseudomonas* Agar Base supplemented with Cetrimide, Fucidin, and Cephaloridine was selected for *Pseudomonas aeruginosa* colony enumeration (ISO13270). Fungal strains were enumerated on yeast extract peptone dextrose agar medium supplemented with 0.05 g/L of chloramphenicol.

### 2.2 Methods

#### 2.2.1 Experimental methods

**Figure 1:**
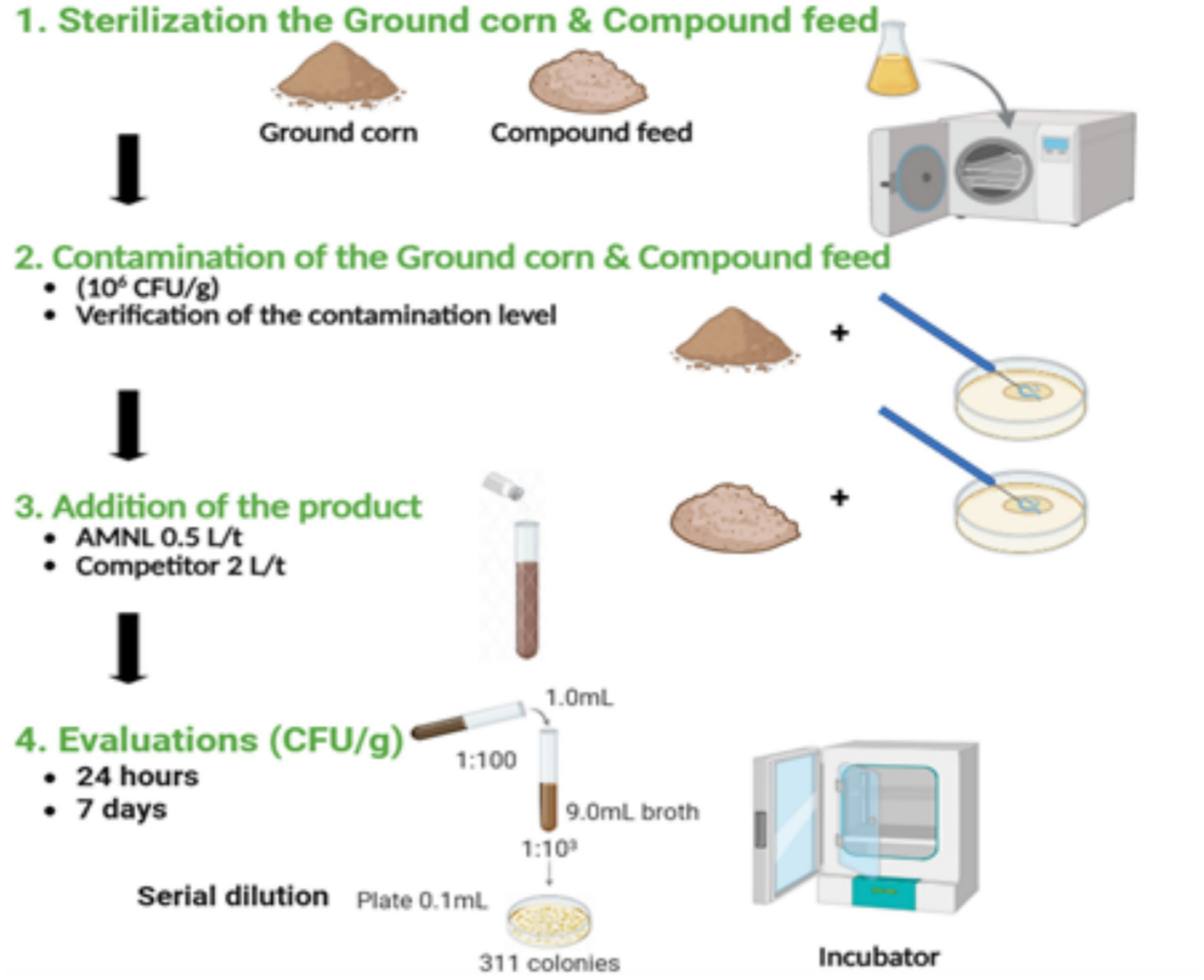
Schematic overview of feed sterilization, artificial contamination (10^6^ CFU/g), product addition, and microbial evaluation by serial dilution and plate counting after 24 h and 7 days (CFU/g).

The experimental procedure for each microorganism and both substrates (compound feed and ground corn) is demonstrated in Figure 1.

The compound feed and ground corn were sterilized at 121 °C for 15 min. Substrates were cooled to room temperature before inoculation. All samples were prepared under aseptic conditions. The AMNL treatment was applied at a concentration of 0.5 L/ton, while the competitor was applied at 2 L/ton, following the recommendation of the manufacturer. All test samples were standardized to 10^6^ CFU/g for each organism by adding 100 μL of a 10^8^ CFU/mL suspension to 10 g of substrate. For the treatment test, bacterial samples were incubated at 37 °C, which represents the optimal growth temperature for mesophilic bacteria. Fungal samples were cultivated at 28 °C, a temperature commonly used for the cultivation of filamentous fungi. These conditions were selected to promote active microbial growth and enable assessment of antimicrobial efficacy under favorable conditions. The fungal incubation temperature represents typical ambient storage conditions, whereas the bacterial incubation temperature reflects an optimal growth environment. Positive controls consisted of 10 g of each substrate by adding 100 μL of a 10^8^ CFU/mL suspension, without treatment (Table 1). Sampling was performed at 24 h and 7 days to evaluate both short-term and long-term antimicrobial effects. Experiments were conducted in triplicate for each combination of microorganism, substrate, treatment, and time point. Each replicate was prepared independently. Treatments were applied by uniformly mixing the appropriate volume of product into the substrate to ensure homogeneous distribution. Water activity (aw) and relative humidity were not directly measured; however, all samples were prepared and incubated under identical conditions to ensure consistency across treatments.

**Table 1:** Formulation of samples in this study (*n* = 3).

Samples	Feed types	Organisms	Treatments
Treated	Compound feed 10 g	Inoculated 10^7^ CFU	AMNL 0.5 L/ton
		Inoculated 10^7^ CFU	Competitor 2 L/ton
	Ground corn 10 g	Inoculated 10^7^ CFU	AMNL 0.5 L/ton
		Inoculated 10^7^ CFU	Competitor 2 L/ton
Control	Compound feed 10 g	Inoculated 10^7^ CFU	None
	Ground corn 10 g	Inoculated 10^7^ CFU	None

Microbial counts were determined in colony-forming units per gram (CFU/g) following serial dilution and plating. Values were also log-transformed for statistical analysis. CFU reduction was calculated as the difference between the control and treated samples (Equation 1). Log reduction was calculated as the difference between the log₁₀-transformed values (Equation 2). CFU values were used to illustrate the magnitude of reduction, while log-transformed values were used for statistical analysis.

CFUReduction=CFUcontrol−CFUtreated (1)

logReduction=log(CFUcontrol)−log(CFUtreated) (2)

#### 2.2.2 Statistical analysis

All statistical analyses were performed using R software (version 4.5.2). All data are presented as the mean ± standard deviation (SD) based on triplicate measurements. Microbial counts were expressed as log-transformed values (log CFU/g) before analysis to improve normality and stabilize variance. Differences between treatments (AMNL vs. competitor) at individual time points and within each feed matrix were evaluated using Welch’s *t*-test, which does not assume equal variances between groups. Independence of observations was ensured by experimental design. Normality of the data distribution was assessed using Shapiro–Wilk tests and visual inspection of Q–Q plots.

To evaluate the overall effects of treatment and time, linear mixed-effects models (LMMs) were fitted using treatment and time as fixed effects. Microbial species were included as a random effect to account for interspecies variability in responses. Models were fitted in R using the lme4 package. Model assumptions were assessed using diagnostic procedures, including the visual inspection of residual-versus-fitted-value plots to evaluate homoscedasticity and Q–Q plots of residuals to assess normality. No substantial deviations from model assumptions were observed. Model estimates are reported with corresponding *p*-values, and statistical significance was defined at *p < 0.05*. Estimated marginal means (EMMs) were calculated from the linear mixed-effects models to provide adjusted mean values for each treatment and time point, accounting for the variability associated with random effects. EMMs allow comparison of treatment effects independent of differences between microbial species.

## 3 Results

### 3.1 AMNL exhibited greater antibacterial and antifungal ability

**Figure 2:**
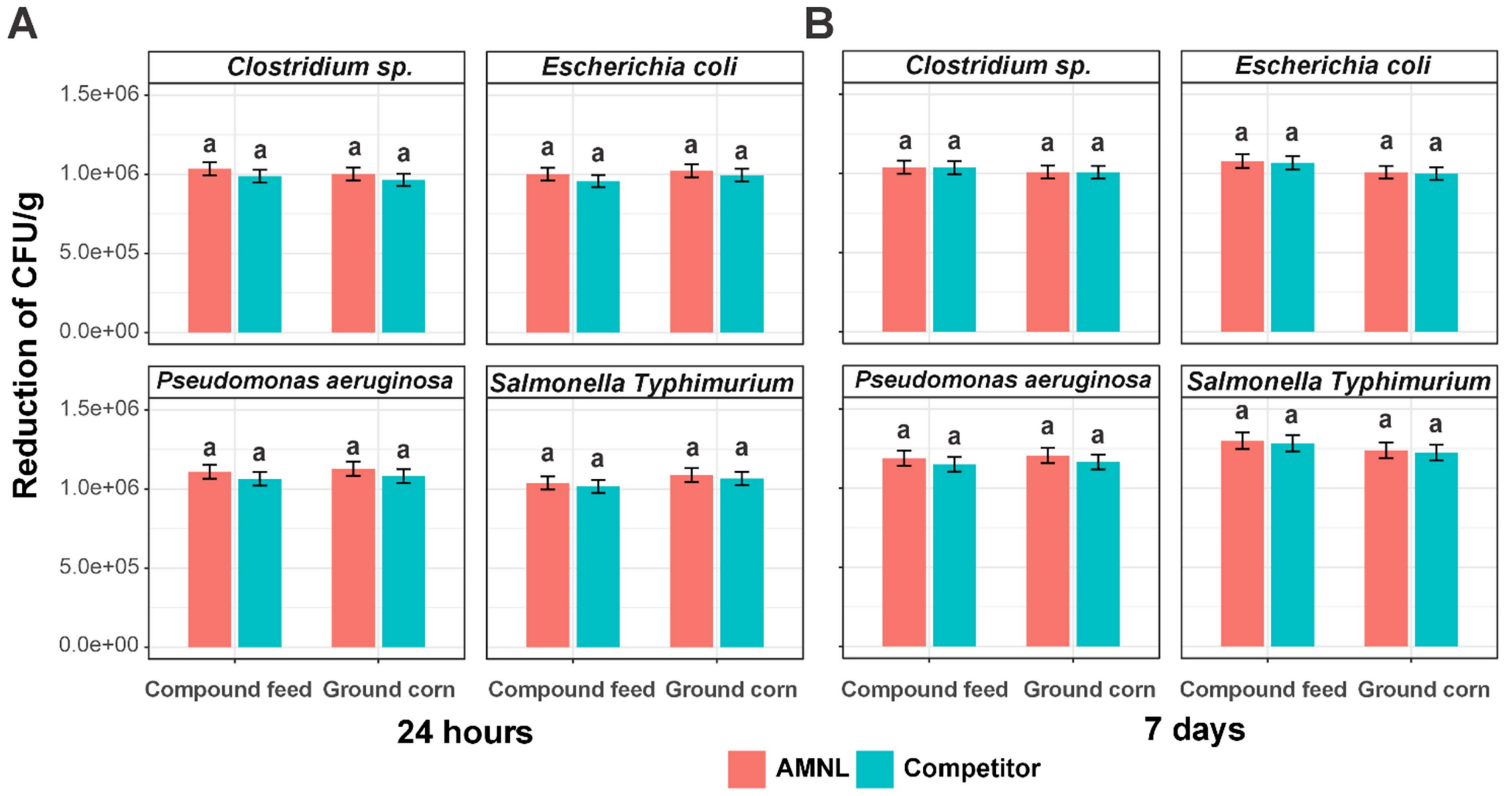
Reduction of bacterial CFU/g by AMNL and competitor over time (*n* = 3). **(A)** 24 h; **(B)** 7 days. Lowercase letters represent the significant differences in reduced bacterial cells between AMNL and competitor in one feed type product (Welch *t*-test *p* < 0.05). Error bars represent standard deviation. Data are presented by microorganisms and are not pooled across taxa.

The reduction in bacterial CFU/g for each microorganism across compound feed and ground corn was observed after 24 h and 7 days (Figure 2). AMNL generally showed greater reductions than the competitor across most bacterial taxa and feed matrices. As shown in Figure 2A, the CFU/g of four bacteria, *Clostridium* sp., *Escherichia coli*, *Pseudomonas aeruginosa,* and *Salmonella Typhimurium*, treated by AMNL were more reduced than the competitor in both compound feed and ground corn in 24 h. On average, AMNL showed a greater absolute bacterial reduction than the competitor by 3.64 × 10^4^ ± 1.12 × 10^4^ number of cells after 24 h (Supplementary Table S1). After 7 days of treatment, both AMNL and the competitor inhibited more cells than after 24 h of treatment. They did not show any significant differences in bacterial cell numbers across feed type products (Figure 2B). On average, the reduced cell numbers treated with AMNL were 1.64 × 10^4^ ± 1.52 × 10^4^ higher than those of competitors within 7 days (Supplementary Table S1).

**Figure 3:**
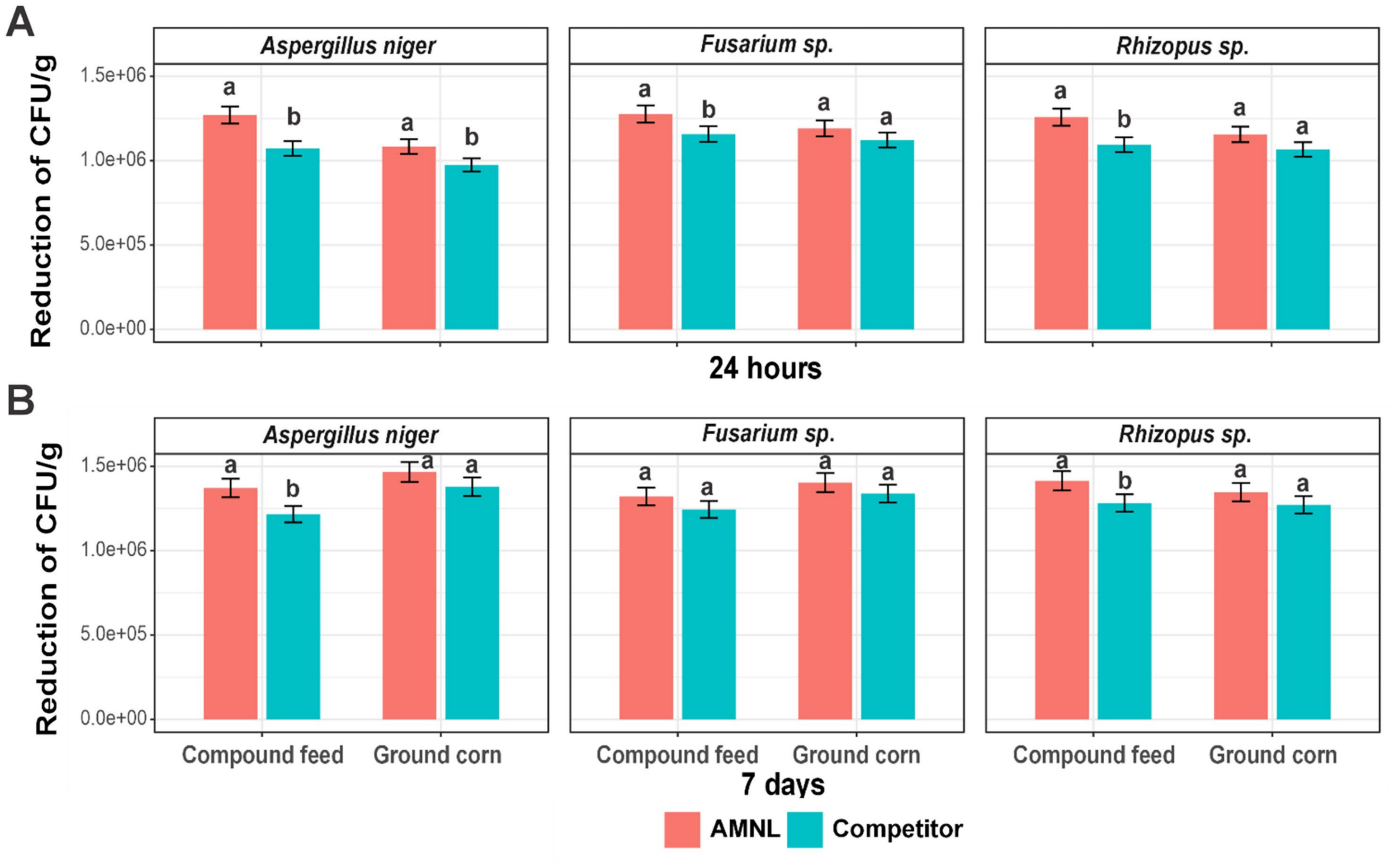
Reduction of fungal CFU/g by AMNL and competitor over time (*n* = 3). **(A)** 24 h; **(B)** 7 days. Lowercase letters represent the significant differences of reduced fungal cells between AMNL and competitor in one feed type product (Welch’s *t*-test *p* < 0.05). Error bars represent standard deviation. Microorganisms present data and are not pooled across taxa.

AMNL performed better antifungal inhibition capacity than the competitor. AMNL significantly hindered significantly higher number of cells than competitor for *Aspergillus niger* in both compound feed and ground corn (Figure 3A). For *Fusarium* sp. and *Rhizopus* sp., AMNL had a better inhibition than competitor in compound feed (Figure 3A). In 24-h treatment for compound feed and ground corn, AMNL prevented more than 1.24 × 10^5^ ± 4.80 × 10^4^ fungal cells than competitor in feed products after 24 h (Supplementary Table S2). Whereas AMNL displayed the significant inhibition ability for *Aspergillus niger* and *Rhizopus* sp. in compound feed after 7 days (Figure 3B). AMNL reduced more than 9.87 × 10^4^ ± 3.62 × 10^4^ fungal cells after 7 days of treatment (Supplementary Table S2). Overall, AMNL demonstrated better antibacterial and antifungal capacity in compound feed and ground corn after short- and long-term treatments. Especially for *Aspergillus niger* and *Rhizopus* sp. in compound feed, AMNL possessed the highest inhibition capacity.

### 3.2 Long-term treatment improved the bacterial and fungal inhibition in different feed type products

**Figure 4:**
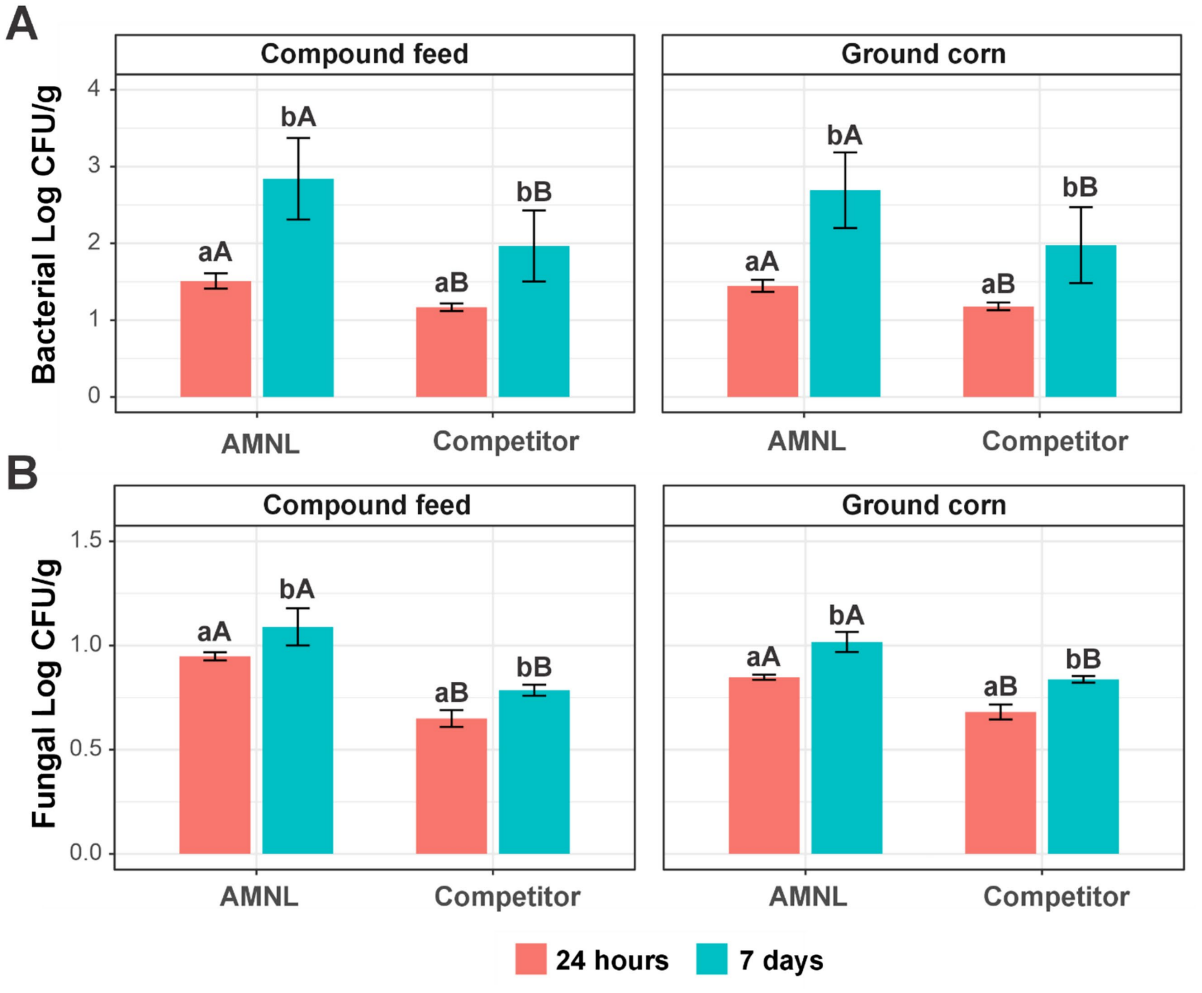
Log CFU/g reduction of microbial counts pooled across taxa (*n* = 3). Values represent mean log reductions across microorganisms for each treatment and time point. **(A)** Reduced bacterial log CFU/g; **(B)** reduced fungal log CFU/g. Lowercase letters represent the significant differences of reduced cells by AMNL or competitor between 24 h and 7 days (Welch *t*-test *p* < 0.05); capital letters represent the significant differences of reduced cells at the same time point between AMNL and competitor (Welch *t*-test *p* < 0.05). Error bars represent standard deviation.

Presenting organism-specific results allowed for the evaluation of variability in treatment response across microbial taxa, while pooled analyses provided an overall assessment of treatment effects across microorganisms. Bacterial log CFU/g reduction tended to be higher at 7 days compared to 24 h. However, these differences were not consistently evident in pairwise comparisons under individual conditions (Figure 4A). When comparing products across feed types, AMNL demonstrated significantly greater bacterial inhibition than the competitor in both compound feed and ground corn. Similarly, fungal log CFU/g reduction showed some variation between 24 h and 7 days, with more pronounced differences observed at 24 h under certain conditions (Figure 4B). Across both feed matrices, AMNL has generally demonstrated greater antifungal efficacy than its competitor.

### 3.3 AMNL showed significantly greater bacterial and fungal reduction than competitor

**Figure 5:**
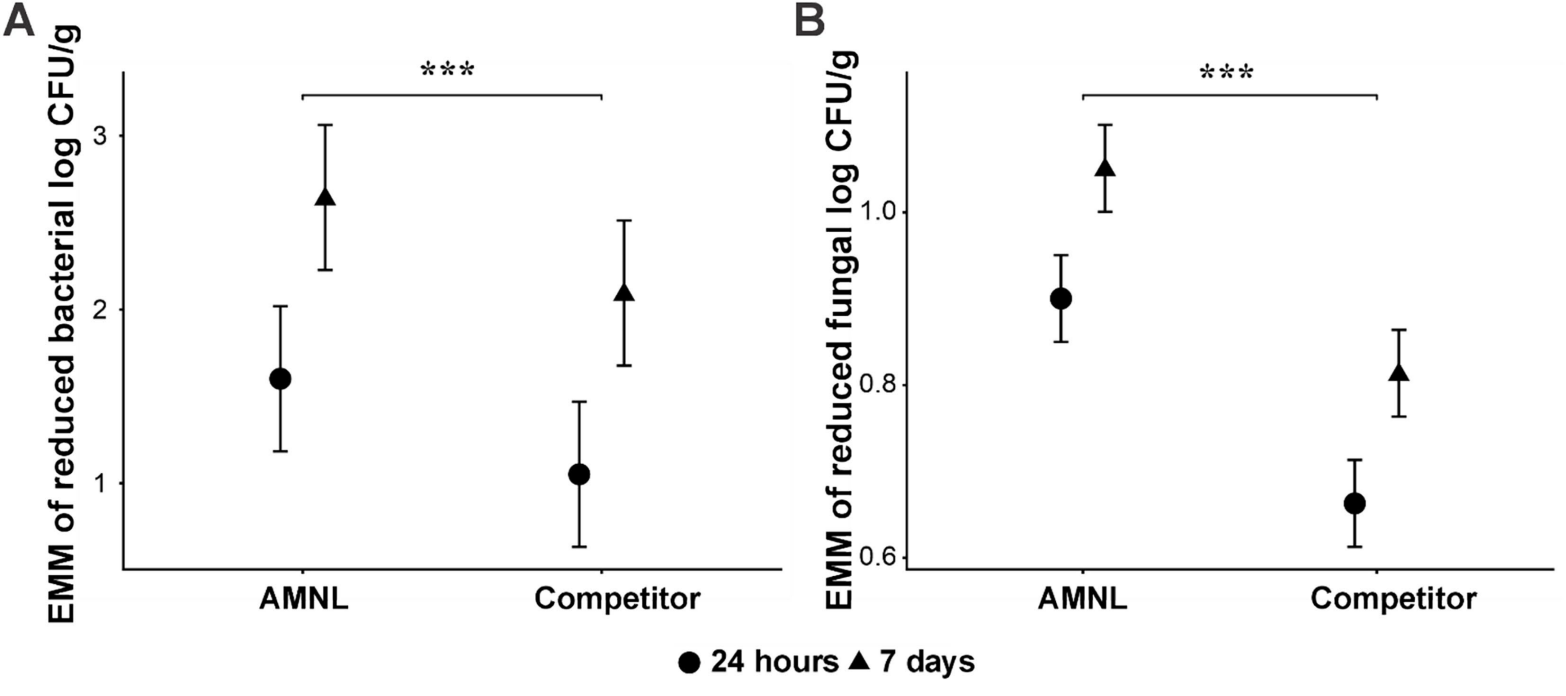
Estimated marginal means (EMMs) of microbial log CFU/g reduction derived from the linear mixed-effects model (*n* = 3). **(A)** Bacterial model; **(B)** fungal model. Values represent model-adjusted means pooled across taxa, with microorganism included as a random effect. Statistical significance for treatment and time effects is reported in the main text from the model coefficients. Error bars represent standard deviation.

Across the compound feed and ground corn matrices, the linear mixed-effects model identified the significant main effects of treatment and time on bacterial log CFU/g reduction (Figure 5A). The corresponding estimated marginal means (EMMs) are presented, and the *p*-values and coefficients reported below were derived from the model output. Relative to AMNL, the competitor exhibited significantly lower bacterial reduction (estimate = −0.55 log, *p* = 0.0099). Bacterial reduction was also significantly greater at 7 days than at 24 h (estimate = +1.04, *p* < 0.001). The inclusion of the organism as a random effect accounted for interspecies variability in baseline response while confirming consistent treatment effects across bacterial taxa. No significant product × time interaction was detected, indicating that product performance was stable over time.

When fungal data were pooled across feed types, significant main effects of product and time were also observed (Figure 5B). The competitor produced significantly lower fungal reduction than AMNL (estimate = −0.24 log, *p* < 0.001), and reductions were modestly but significantly greater at 7 days compared to 24 h (estimate = +0.15 log, *p* < 0.001). Between-organism variance was negligible, indicating a uniform fungal response across species. As with bacteria, no evidence of a product × time interaction was observed.

Collectively, these results demonstrated that AMNL consistently showed a trend toward higher reductions than the competitor across microbial groups, feed matrices, and time points. Time strongly influenced bacterial reduction and exerted a smaller but significant effect on fungal reduction. Treatment effects were robust to organismal variation and did not differ over time, supporting the broad and stable antimicrobial efficacy of AMNL.

## 4 Discussion

The antimicrobial and antifungal activities of AMNL (0.5 L/ton) and the competitor product (2 L/ton) were evaluated in ground corn and compound feed against selected bacterial and fungal contaminants. AMNL comprises citric acid and phenolic compounds, both of which are recognized for their antimicrobial effects (Figures 2, 3).

The observed antimicrobial effects may be partly attributed to the action of organic acids, which are known to disrupt intracellular pH homeostasis and metabolic activity (Mani-López et al., 2012; Dubal et al., 2004). Previous studies have reported the use of propionic and acetic acids at concentrations between 0.5 and 2% for microbial control in feed and food systems (Mani-López et al., 2012; Dubal et al., 2004; Huang and Chen, 2011; Park et al., 2011). The 1% citric acid content in AMNL is therefore within a comparable range, although differences in antimicrobial performance may arise from variations in acid dissociation, membrane permeability, and interactions with other formulation components.

Phenolic compounds, such as thymol, are known to disrupt microbial membranes and increase permeability, which may contribute to the reductions observed in this study (Riella et al., 2012; Sancheti et al., 2013; Marchese et al., 2016; Parsaei et al., 2016). In addition to thymol, AMNL contains cineole and cymene, which are known to enhance antimicrobial efficacy through membrane disruption and permeability modulation. Previous studies have reported potential synergistic interactions between organic acids and phenolic compounds; however, such interactions were not directly evaluated in the present study (Mączka et al., 2023). As illustrated in Figure 4, a tendency for increased microbial reduction over time was observed under some conditions. After 7 days, the antimicrobial efficacy of the tested preservatives was higher than after 24 h, a trend consistent with findings on natural preservatives, such as *Thymus zygis* extracts (Coimbra et al., 2022). The efficacy of thymol against *Listeria* strains has been shown to persist for more than 14 days in various food models. However, the food matrix significantly influences thymol activity: proteins and lipids typically exert a negative impact, whereas carbohydrates have a positive effect on efficacy (Machado et al., 2017).

Previous research has demonstrated that thymol exhibits enhanced antimicrobial efficacy when combined with complementary monoterpenes, resulting in synergistic activity against pathogens such as *Escherichia coli*, *Staphylococcus aureus*, and *Vibrio cholerae* (Xu et al., 2008; Rattanachaikunsopon and Phumkhachorn, 2010; Rúa et al., 2019). Furthermore, low concentrations of this phenol are beneficial for maintaining the organoleptic properties of the feed (Rúa et al., 2019). Consequently, AMNL achieves superior antimicrobial capacity even at a low application volume (0.5 L/ton).

The stronger temporal response observed in bacteria than in fungi may reflect differences in susceptibility, although this was not directly investigated. Fungal reductions were more uniform across species and time, consistent with their greater structural resilience. Importantly, the efficacy of AMNL was unaffected by the feed matrix, suggesting that its antimicrobial activity remains stable despite the varying substrate complexity of compound feed and ground corn.

The greater antimicrobial activity observed for AMNL may be related to differences in formulation composition and interactions with the feed matrix (Figure 5). Significant effects of time were identified using the linear mixed-effects model, whereas the random-effects component of the model confirmed that treatment effects were consistent across microbial taxa, supporting a broad-spectrum mode of action. Collectively, these findings suggest that AMNL may serve as a promising antimicrobial feed additive under the conditions tested.

Limitations of this study include that the individual contributions of citric acid and phenolic compounds were not evaluated separately; additionally, initial inoculum levels were not immediately confirmed. Therefore, potential synergistic interactions between these components cannot be confirmed. Future studies using factorial experimental designs are needed to assess the presence and magnitude of such interactions.

## 5 Conclusion

This study indicates that Alquermold Natural Plus L (AMNL) provides effective microbial control in compound feed and ground corn under the conditions tested. AMNL generally showed greater antimicrobial activity than a commercial preservative against selected bacterial and fungal contaminants, despite being applied at a lower inclusion rate. Antimicrobial activity increased over the storage period evaluated, while no major differences were observed between the feed matrices. This formulation, based on citric acid and phenolic compounds, may contribute to efficient preservation at low inclusion levels. However, potential synergistic interactions were not directly assessed in this study. Overall, AMNL represents a promising natural alternative to improve feed safety and shelf life, although further studies under a wide range of conditions are needed to confirm these findings.

## Data Availability

The original contributions presented in the study are included in the article/Supplementary material; further inquiries can be directed to the corresponding author.
