# Supplementary material for: Broad-spectrum antimicrobial activity of a citric acid–phenolic formulation in animal feed matrices
Source: Front Microbiol. 2026 May 4;17:1822735. doi: 10.3389/fmicb.2026.1822735 (PMC13180739; doi:10.3389/fmicb.2026.1822735)
Supplement: Supplementary file 1 [file Table_1.docx]

| **Table S1.** The bacterial CFU/g in samples by treatments after 24 hours and 7 days (n = 3) | | | | | | |
| --- | --- | --- | --- | --- | --- | --- |
| **Organisms** | **Feed types** | **Treatments** | **24 hours** | | **7 Days** | |
|  |  |  | **Reduced CFU/g** | **Δ Reduced CFU/g** | **Reduced CFU/g** | **Δ Reduced CFU/g** |
| *E. coli* | Ground corn | AMNL | 1.02 × 10^6^ ± 4.09 × 10^4^ | 2.70 × 10^4^ ± 1080 | 1.01 × 10^6^ ± 4.03 × 10^4^ | 0.86 × 10^4^ ± 344 |
|  | Ground corn | Competitor | 0.99 × 10^6^ ± 3.99 × 10^4^ |  | 0.99 × 10^6^ ± 3.99 × 10^4^ |  |
|  | Compound feed | AMNL | 1.00 × 10^6^ ± 3.98× 10^4^ | 4.40 × 10^4^ ± 1760 | 1.08 × 10^6^ ± 4.31 × 10^4^ | 1.12 × 10^4^ ± 448 |
|  | Compound feed | Competitor | 0.96 × 10^6^ ± 3.81 × 10^4^ |  | 1.07 × 10^6^ ± 4.27 × 10^4^ |  |
| *S. Typhimurium* | Ground corn | AMNL | 1.09 × 10^6^ ± 4.35 × 10^4^ | 2.17 × 10^4^ ± 868 | 1.24 × 10^6^ ± 4.96 × 10^4^ | 1.39 × 10^4^ ± 559 |
|  | Ground corn | Competitor | 1.07 × 10^6^ ± 4.26 × 10^4^ |  | 1.23 × 10^6^ ± 4.90 × 10^4^ |  |
|  | Compound feed | AMNL | 1.04 × 10^6^ ± 4.15 × 10^4^ | 2.17 × 10^4^ ± 686 | 1.30 × 10^6^ ± 5.20 × 10^4^ | 1.50 × 10^4^ ± 601 |
|  | Compound feed | Competitor | 1.01 × 10^6^ ± 4.06 × 10^4^ |  | 1.28 × 10^6^ ± 5.14 × 10^4^ |  |
| *P. aeruginosa* | Ground corn | AMNL | 1.13 × 10^6^ ± 4.51 × 10^4^ | 4.74 × 10^4^ ± 1896 | 1.21 × 10^6^ ± 4.83 × 10^4^ | 4.11 × 10^4^ ± 1644 |
|  | Ground corn | Competitor | 1.08× 10^6^ ± 4.33 × 10^4^ |  | 1.17 × 10^6^ ± 4.66 × 10^4^ |  |
|  | Compound feed | AMNL | 1.11 × 10^6^ ± 4.44 × 10^4^ | 4.57 × 10^4^ ± 1828 | 1.19 × 10^6^ ± 4.76 × 10^4^ | 3.82 × 10^4^ ± 1528 |
|  | Compound feed | Competitor | 1.06 × 10^6^ ± 4.26 × 10^4^ |  | 1.15 × 10^6^ ± 4.60 × 10^4^ |  |
| *Clostridium sp.* | Ground corn | AMNL | 1.00 × 10^6^ ± 4.01 × 10^4^ | 3.75 × 10^4^ ± 1500 | 1.01 × 10^6^ ± 4.04 × 10^4^ | 0.12 × 10^4^ ± 49 |
|  | Ground corn | Competitor | 0.96 × 10^6^ ± 3.86 × 10^4^ |  | 1.01 × 10^6^ ± 4.03 × 10^4^ |  |
|  | Compound feed | AMNL | 1.04 × 10^6^ ± 4.14 × 10^4^ | 4.65 × 10^4^ ± 1860 | 1.04 × 10^6^ ± 4.16 × 10^4^ | 0.17 × 10^4^ ± 69 |
|  | Compound feed | Competitor | 0.99 × 10^6^ ± 3.96 × 10^4^ |  | 1.04 × 10^6^ ± 4.15 × 10^4^ |  |
|  |  |  | **Avg.** | 3.64 × 10^4^ ± 1.12 × 10^4^ |  | 1.64 × 10^4^ ± 1.52 × 10^4^ |
