# Supplementary material for: Broad-spectrum antimicrobial activity of a citric acid–phenolic formulation in animal feed matrices
Source: Front Microbiol. 2026 May 4;17:1822735. doi: 10.3389/fmicb.2026.1822735 (PMC13180739; doi:10.3389/fmicb.2026.1822735)
Supplement: Supplementary file 2 [file Table_2.docx]

| **Table S2.** The fungal CFU/g in samples by treatments after 24 hours and 7 days (n = 3) | | | | | | |
| --- | --- | --- | --- | --- | --- | --- |
| **Organisms** | **Feed types** | **Treatments** | **24 hours** | | **7 Days** | |
|  |  |  | **Reduced CFU/g** | **Δ Reduced CFU/g** | **Reduced CFU/g** | **Δ Reduced CFU/g** |
| *A. niger* | Ground corn | AMNL | 1.08 × 10^6^ ± 4.34 × 10^4^ | 1.08 × 10^5^ ± 4320 | 1.47 × 10^6^ ± 5.87 × 10^4^ | 0.88 × 10^5^ ± 3520 |
|  | Ground corn | Competitor | 0.98 × 10^6^ ± 3.90 × 10^4^ |  | 1.38 × 10^6^ ± 5.52 × 10^4^ |  |
|  | Compound feed | AMNL | 1.27 × 10^6^ ± 5.08× 10^4^ | 1.98 × 10^5^ ± 7920 | 1.37 × 10^6^ ± 5.49 × 10^4^ | 1.55 × 10^5^ ± 6200 |
|  | Compound feed | Competitor | 1.07 × 10^6^ ± 4.29 × 10^4^ |  | 1.22 × 10^6^ ± 4.87 × 10^4^ |  |
| *Rhizopus sp.* | Ground corn | AMNL | 1.16 × 10^6^ ± 4.62 × 10^4^ | 0.89 × 10^5^ ± 3560 | 1.35 × 10^6^ ± 5.39 × 10^4^ | 0.75 × 10^5^ ± 3000 |
|  | Ground corn | Competitor | 1.07 × 10^6^ ± 4.27 × 10^4^ |  | 1.27 × 10^6^ ± 5.09 × 10^4^ |  |
|  | Compound feed | AMNL | 1.26× 10^6^ ± 5.03 × 10^4^ | 1.63 × 10^5^ ± 6520 | 1.42 × 10^6^ ± 5.67 × 10^4^ | 1.32 × 10^5^ ± 5280 |
|  | Compound feed | Competitor | 1.10 × 10^6^ ± 4.38 × 10^4^ |  | 1.28 × 10^6^ ± 5.13 × 10^4^ |  |
| *Fusarium sp.* | Ground corn | AMNL | 1.19 × 10^6^ ± 4.77 × 10^4^ | 0.69 × 10^5^ ± 2760 | 1.40 × 10^6^ ± 5.62 × 10^4^ | 0.65 × 10^5^ ± 2600 |
|  | Ground corn | Competitor | 1.12× 10^6^ ± 4.49 × 10^4^ |  | 1.34 × 10^6^ ± 5.36 × 10^4^ |  |
|  | Compound feed | AMNL | 1.28 × 10^6^ ± 5.11 × 10^4^ | 1.19 × 10^5^ ± 4760 | 1.32 × 10^6^ ± 5.29 × 10^4^ | 0.77 × 10^5^ ± 3080 |
|  | Compound feed | Competitor | 1.16 × 10^6^ ± 4.63 × 10^4^ |  | 1.25 × 10^6^ ± 4.98 × 10^4^ |  |
|  |  |  | **Avg.** | 1.24 × 10^5^ ± 4.80 × 10^4^ |  | 9.87 × 10^4^ ± 3.62 × 10^4^ |
